# Quantifying the burden of disease due to premature mortality in Hong Kong using standard expected years of life lost

**DOI:** 10.1186/1471-2458-13-863

**Published:** 2013-09-18

**Authors:** Dietrich Plass, Patsy Yuen Kwan Chau, Thuan Quoc Thach, Heiko J Jahn, Poh Chin Lai, Chit Ming Wong, Alexander Kraemer

**Affiliations:** 1 Department of Public Health Medicine, School of Public Health, Bielefeld University, Bielefeld, Germany; 2 School of Public Health, The University of Hong Kong, Hong Kong SAR, China; 3 Department of Geography, The University of Hong Kong, Hong Kong SAR, China

**Keywords:** Burden of disease, Hong Kong, Standard expected years of life lost, SEYLL

## Abstract

**Background:**

To complement available information on mortality in a population Standard Expected Years of Life Lost (SEYLL), an indicator of premature mortality, is increasingly used to calculate the mortality-associated disease burden. SEYLL consider the age at death and therefore allow a more accurate view on mortality patterns as compared to routinely used measures (e.g. death counts). This study provides a comprehensive assessment of disease and injury SEYLL for Hong Kong in 2010.

**Methods:**

To estimate the SEYLL, life-expectancy at birth was set according to the 2004 Global Burden of Disease study at 82.5 and 80 years for females and males, respectively. Cause of death data for 2010 were corrected for misclassification of cardiovascular and cancer causes. In addition to the baseline estimates, scenario analyses were performed using alternative assumptions on life-expectancy (Hong Kong standard life-expectancy), time-discounting and age-weighting. To estimate a trend of premature mortality a time-series analysis from 2001 to 2010 was conducted.

**Results:**

In 2010 524,706.5 years were lost due to premature death in Hong Kong with 58.3% of the SEYLL attributable to male deaths. The three overall leading single causes of SEYLL were “trachea, bronchus and lung cancers”, “ischaemic heart disease” and “lower respiratory infections” together accounting for about 29% of the overall SEYLL. Further, self-inflicted injuries (5.6%; ranked 5) and liver cancer (4.9%; ranked 7) were identified as important causes not adequately captured by classical mortality measures. Scenario analyses highlighted that by using a 3% time-discount rate and non-uniform age-weights the SEYLL dropped by 51.6%. Using Hong Kong’s standard life-expectancy values resulted in an overall increase of SEYLL by 10.8% as compared to the baseline SEYLL. Time-series analysis indicates an overall increase of SEYLL by 6.4%. In particular, group I (communicable, maternal, perinatal and nutritional) conditions showed highest increases with SEYLL-rates per 100,000 in 2010 being 1.4 times higher than 2001.

**Conclusions:**

The study stresses the mortality impact of diseases and injuries that occur in earlier stages of life and thus presents the SEYLL measure as a more sensitive indicator compared to classical mortality indicators. SEYLL provide useful additional information and supplement available death statistics.

## Background

All over the world, and not only since the impact of the global financial crisis in 2008, resources in the health care sector are scarce [1,2]. Epidemiological indicators such as mortality patterns and life-expectancy values, derived from historically observed mortality trends, have been used extensively to describe and quantify health improvements on population level and to set priorities for resource allocation [3,4]. Classical mortality indicators are strongly influenced by diseases where death occurs at older ages and thus do not represent the complete mortality-associated disease burden. Indicators that take into account age at death allow a more comprehensive assessment of mortality because they not only count the number of deaths but also measure the years of life lost due to premature mortality [5-7]. Health indicators using lost time as an outcome are increasingly used in public health at global, national and local levels [8-12]. Many of these measures combine complementary epidemiologic information and present the health of a population as a single numerical indicator. Such health indicators are categorised as summary measures of population health [13]. The disability-adjusted life year (DALY) has frequently been used in population health assessments [14]. The DALY combines the impact of mortality and morbidity and uses time as the unit to measure health losses in populations [15]. It is presented as the sum of years lived with disability (YLD, morbidity component) and years of life lost due to premature death (YLL, mortality component). Owing to often poor or even unavailable detailed information on non-fatal outcomes [16], several investigations have focused on the calculation of single DALY components [5-7,17-21]. Most of these studies used data from vital statistics where the quality and availability of data have improved in the past to calculate years lost due to premature death. One commonly used measure of premature death is the standard expected years of life lost (SEYLL) indicator. The SEYLL, as a standardised measure, can be used to quantify and compare the impact of different diseases and injuries. SEYLL can supplement the traditional set of mortality indicators and enhance the monitoring of disease patterns in populations.

Currently, no comprehensive assessment of premature mortality effects is available for the Special Administrative Region of Hong Kong. Therefore, the major objective of this study is to quantify the SEYLL due to premature mortality in Hong Kong for the year 2010. Secondary objectives are to estimate the effect of different social value choices and to present the changes of mortality associated disease burden from 2001 to 2010.

## Methods

### Standard expected years of life lost

Methods provided by the Global Burden of Disease (GBD) framework were adopted for the Hong Kong setting and SEYLL were quantified by cause, sex and 19 age-groups for the year 2010 [22]. The baseline-SEYLL were calculated by multiplying the number of deaths *(N)* at a certain age of death with the remaining life-expectancy *(L)* at age of death *(x)*:

$$\mathrm{SEYLL}=N\times L_{x}$$

Introducing age-weighting and time-discounting, the formula results in the following term:

$$\begin{array}{l} \text{SEYLL}=N\;Ce^{\left(\mathrm{ra}\right)}/\left(\beta +r\right)²[e^{-\left(\beta +r\right)\left(L+a\right)}\left[-\left(\beta +r\right)\left(L+a\right)-1\right]\\ \;-e^{-\left(\beta +r\right)a}\left[-\left(\beta +r\right)a-1\right][ \end{array}$$

[23]

where *N* is the number of deaths, *r* is the discount rate (0.03), *C* is the age-weighting correction constant (0.1658), *e* is a constant (≈ 2.718), *β* is the parameter from the age-weighting function (0.04), *a* is the age at death and *L* the remaining life-expectancy at age of death [23].

The diseases and injuries are classified according to the GBD classification system and ordered by four levels of disaggregation [9]. At the first level, conditions are presented in three broad groups, namely communicable, maternal, perinatal and nutritional conditions (group I), non-communicable conditions (group II) and injuries (group III). For detailed analyses, these broad groups are differentiated into 21 condition groupings and then further into more than 100 single disease entities. A normative health goal was set, defining life-expectancy according to a World Health Organisation (WHO) standard at 82.5 and 80 years for women and men, respectively [14]. SEYLL-rates per 100,000 population are calculated using the age and sex stratified mid-year population for Hong Kong in 2010 [24]. SEYLL results are reported with uniform age-weighting and no time-discount [25-27]. Sensitivity analyses were performed using local standard life-expectancy values for the Hong Kong population and including different age-weighting and time-discounting scenarios.

### Data

Population data for 2010 were obtained from the HK Government Department with a mid-year population of 7.07 million inhabitants [28].

Death counts for 2010 were extracted from a dataset provided by the Hong Kong Census and Statistics Department [29]. This dataset contained information about cause of death, age and sex of each deceased. The causes of death were classified according to the tenth revision of the International Classification of Diseases (ICD-10). Deaths classified as S00 to T98 (Injury, poisoning and certain other consequences of external causes) were separately derived from external causes of death.

The causes of death data were translated to the GBD classification system using the recommended algorithms [30]. In addition, ill-defined causes (R00-R99), cancer (C76, C80, C97) and cardiovascular (I47.2, I49.0, I46, I50, I51.4, I51.5, I51.6, I51.9, I70.9) garbage-codes were redistributed according to correction methods provided by the GBD study [9,31]. A time series analysis of death registry data from 2001 to 2010 was performed to estimate the past trends of SEYLL in Hong Kong.

The calculations were performed using Microsoft Office Excel version 12 and R Statistical Software version 2.15.1.

## Results

### SEYLL by main broad cause groups

During 2010, an overall number of 41,887 (female 44.4%; male 55.6%) deaths were registered in Hong Kong, resulting in 524,706.5 years lost due to premature death, with 41.7% and 58.3% of the SEYLL attributable to female and male deaths, respectively. Overall, the highest percentages of SEYLL were estimated for group II conditions, representing 78.8% of the total disease burden. Group I and III conditions share 12.7% and 8.5%, respectively (Table 1).

**Table 1:** Standard expected years of life lost (SEYLL) by broad cause groups, Hong Kong, 2010

	**Total**			**Female**			**Male**		
**Cause group**	**SEYLL**	**%**	Rate per 100,000*	**SEYLL**	**%**	**Rate per 100,000**	**SEYLL**	**%**	**Rate per 100,000**
**Group I**	66568.5	12.7	947.7	27779.4	12.7	744.8	38789.1	12.7	1177.5
**Group II**	413537.0	78.8	5887.3	174861.2	80.0	4688.1	238675.8	78.0	7245.1
**Group III**	44600.9	8.5	635.0	16054.2	7.3	430.4	28546.7	9.3	866.5
**Total**	524706.5	100.0	7470.0	218694.8	100.0	5863.3	306011.7	100.0	9289.1

Group I: communicable, maternal, perinatal and nutritional conditions.

Group II: non-communicable conditions.

Group III: injuries.

*The rates presented here are rates per 100,000 population (inhabitants of Hong Kong).

The female population showed a higher share for group II conditions, with 80% as compared to the male population (78%). Group I conditions accounted for 12.7% of the SEYLLs for both males and females. Men showed a higher percentage attributable to injuries at 9.3% in comparison to 7.3% for women.

**Figure 1:**
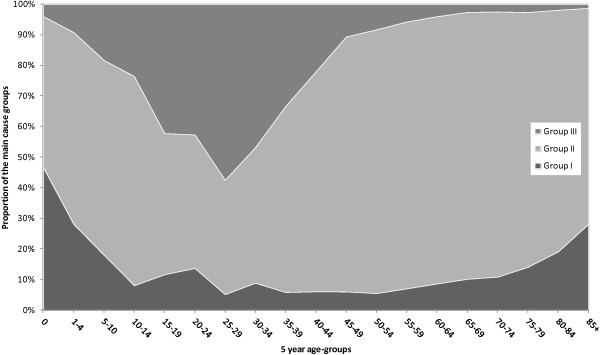
**Distribution of SEYLL by main cause categories and age for Hong Kong, 2010 (both sexes).** Group I: communicable, maternal, perinatal and nutritional conditions. Group II: non-communicable conditions. Group III: injuries.

Figure 1 shows SEYLL shares for the three condition groups stratified by age group. With a share of 46.6% of the total burden at the age group 0–1, group I conditions reach their maximum SEYLL share. The percentage shows a decreasing trend with two smaller peaks of 13.6% and 8.8% in the age-groups 20–24 and 30–34 years, respectively. After a constant phase at about 6%, an increase was identified beginning in the age-group 55–59 years reaching a peak in the age-group 85+ (28.1%).

Group II conditions presented the lowest observed SEYLL shares in the age-group 25–29 years, with 37.4%. The SEYLL due to group II conditions appeared to increase with age, reaching a peak at 87.5% (age-group 60–64).

For group III conditions, a constant rise of the SEYLL share is observed starting in age-group 0, reaching a peak of 57.5% in age-group 25–29 years, dropping rapidly at 45–49 years (10.6%), and then flattening out with increasing age.

**Figure 2:**
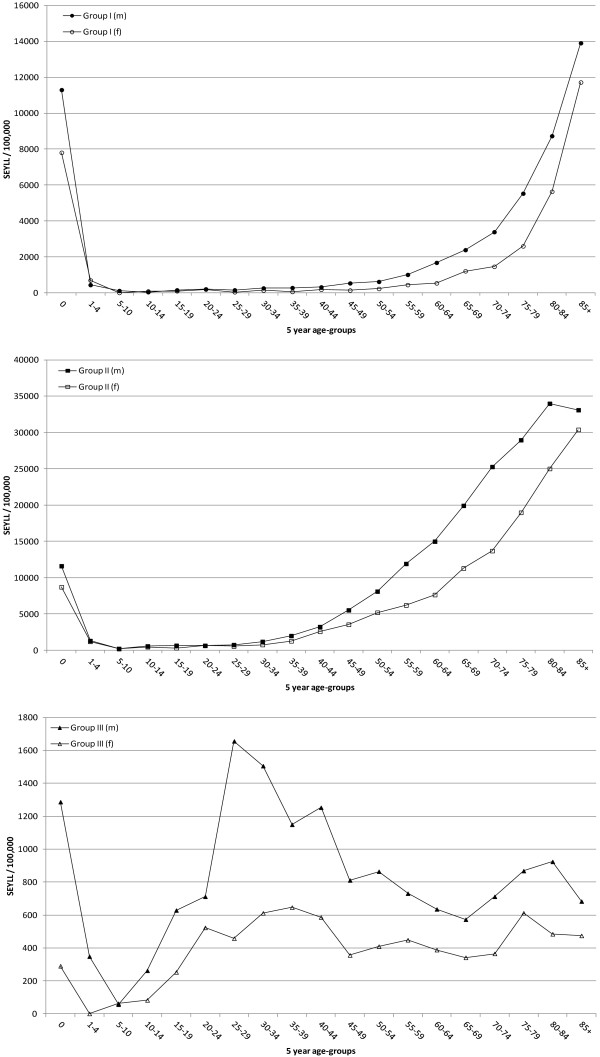
**SEYLL stratified by age-group, sex and the three main cause groups, Hong Kong, 2010.** Group I: communicable, maternal, perinatal and nutritional conditions. Group II: non-communicable conditions. Group III: injuries.

The overall SEYLL-rate per year for the male population (9,289 SEYLL/100,000) exceeded the female rate (5,863 SEYLL/100,000) 1.6-fold. SEYLL-rates for group I and II conditions were higher for the male population, especially in the age-group 0–1 with 11,303.4 SEYLL/100,000 and 11,608.9 SEYLL/100,000 as compared to a female SEYLL-rate of 7,807.5 SEYLL/100,000 and 8,708.3 SEYLL/100,000, respectively (Figure 2). After the age group 0–1 year, group I and II rates for both males and females showed a decreasing trend and remained at a constantly low level followed by an upswing of rates. Even though the rates of group I conditions in the population above 45 years increased for both sexes, the trend for males and females was initially diverging, with male rates presenting a steeper slope, reaching a peak of 13,909.8 SEYLL/100,000 in the age-group 85+, which is 1.2-fold higher than for females (11,724 SEYLL/100,000). For group II conditions, a similar trend could be observed, with very high SEYLL-rates for children aged 0–1 year and subsequent low levels until the age-group of 30–34 years. With further increasing age, the SEYLL-rates for group II conditions rose with a steeper slope for males, reaching a maximum of 34,002.5 SEYLL/100,000 in the age-group 80–84 years. The females showed a maximum of 30,403.3 SEYLL/100,000, which was delayed to the age group of 85+. For all age-groups (except age-group 5–10 years) the male population showed an increased SEYLL-rate due to group III conditions as compared to the female population. The peak was reached at 25–29 years of age with 1,656.7 SEYLL/100,000. In this age group the SEYLL-rate for men was 3.6 times higher than the one estimated for women. SEYLL for group III conditions decreased with age but presented smaller peaks in the age groups 75–79 and 80–84 years for females and males, respectively.

### SEYLL by condition groups

At the first level of disaggregation (Table 2), malignant neoplasms (39.1%), cardiovascular diseases (21.7%) and respiratory infections (8.9%) were the leading cause groups, together accounting for about 70% of the years of life lost due to premature death. Stratification by sex led to similar leading condition groups.

**Table 2:** Standard expected years of life lost (SEYLL) by condition groupings, Hong Kong, 2010

		**Total**				**Female**				**Male**		
**Nr.**	**Condition group**	**SEYLL**	**%**	Rate per 100,000*	**Nr.**	**SEYLL**	**%**	**Rate per 100,000**	**Nr.**	**SEYLL**	**%**	**Rate per 100,000**
**1**	**Malignant neoplasms**	205142.2	39.1	2920.5	1	87801.4	40.1	2354.0	1	117340.8	38.3	3561.9
**2**	**Cardiovascular diseases**	113833.4	21.7	1620.6	2	48034.9	22.0	1287.8	2	65798.5	21.5	1997.3
**3**	**Respiratory infections**	46536.6	8.9	662.5	3	19733.8	9.0	529.1	3	26802.8	8.8	813.6
**4**	**Intentional injuries**	30035.2	5.7	427.6	4	11831.7	5.4	317.2	4	18203.5	5.9	552.6
**5**	**Respiratory diseases**	24050.4	4.6	342.4	6	6979.4	3.2	187.1	5	17071.1	5.6	518.2
**6**	**Genitourinary diseases**	19051.6	3.6	271.2	5	9522.1	4.4	255.3	8	9529.5	3.1	289.3
**7**	**Digestive diseases**	18204.2	3.5	259.2	7	6571.3	3.0	176.2	6	11632.9	3.8	353.1
**8**	**Infectious and parasitic diseases**	15337.7	2.9	218.4	8	6492.2	3.0	174.1	9	8845.5	2.9	268.5
**9**	**Unintentional injuries**	14565.7	2.8	207.4	10	4222.4	1.9	113.2	7	10343.3	3.4	314.0
**10**	**Neuropsychiatric conditions**	11111.0	2.1	158.2	9	5066.8	2.3	135.8	10	6044.3	2.0	183.5

*The rates presented here are rates per 100,000 population (inhabitants of Hong Kong).

**Figure 3:**
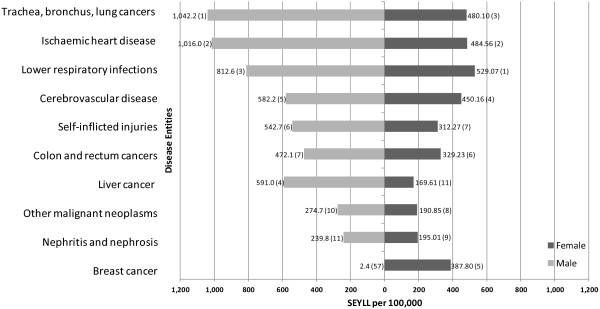
Leading causes of SEYLL by sex and single disease entities, Hong Kong, 2010 (Sex-specific rankings in parentheses).

The top three leading single disease causes of SEYLL were “trachea, bronchus and lung cancers”, “ischaemic heart disease” and “lower respiratory infections”, together accounting for 28.6% of the overall SEYLL in Hong Kong. Stratified by sex, the three leading causes of SEYLL remained the same but differed in their order (Figure 3). For women, lower respiratory infections (529.1 SEYLL/100,000) were the leading cause, followed by ischaemic heart disease (484.6 SEYLL/100,000) and trachea, bronchus and lung cancers (480.1 SEYLL/100,000) (Figure 3). Women were further strongly affected by breast cancer (ranked 5th) and nephritis and nephrosis (ranked 9th), but these entities were not among the ten leading causes of SEYLL for men. The leading cause for men was trachea, bronchus and lung cancers (1,042.3 SEYLL/100,000), followed by ischaemic heart disease (1,016.0 SEYLL/100,000) and lower respiratory infections (812.6 SEYLL/100,000). Furthermore, liver cancer (ranked 4th), chronic obstructive pulmonary disease (ranked 8th) and mouth and oropharynx cancer (ranked 9th) were conditions that were important drivers of SEYLL for men but did not belong to the ten leading causes of SEYLL for women.

The SEYLL-rates for the male population were generally higher for all conditions (Figure 3), especially for ischaemic heart disease, trachea, bronchus and lung cancers, and liver cancer, with rates for males being 2.1, 2.2 and 3.5 times higher than the female rates, respectively. An exception of course was breast cancer, a sex-specific malignant neoplasm mainly occurring in the female population.

The overall leading causes include many malignant neoplasms, which constituted five out of ten leading causes. It is noteworthy that the group III condition self-inflicted injuries strongly affected both sexes and was ranked 6th and 7th in the male and female leading causes, respectively.

**Figure 4:**
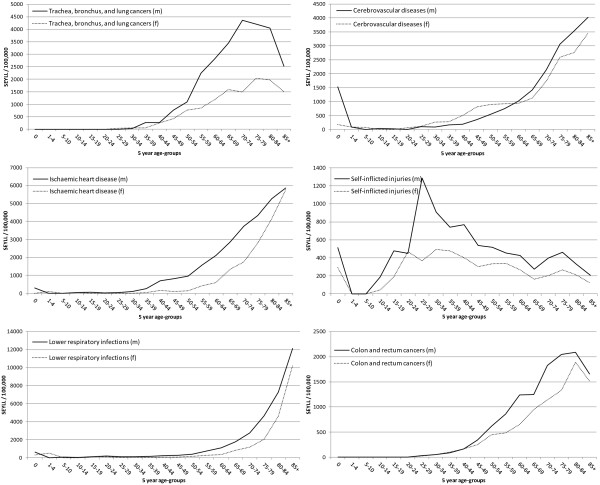
The six leading causes of SEYLL by sex and age-groups, Hong Kong, 2010.

Figure 4 presents the SEYLL for the six leading causes stratified by age and sex, highlighting the age-groups most affected by the presented conditions. Trachea, bronchus and lung cancers showed the highest rates for the population aged above 30 years, reaching a maximum of 4,362.8 SEYLL/100,000 for men aged 70–74 years and 2,043.4 for women aged 75–79 years, with rates being 2.2- to 3-fold higher for the male population. Cerebrovascular and ischaemic heart diseases showed similar trends with a steady increase in SEYLL-rates starting at age 30. A major sex-difference was observed during the age-span between 30–59 years, with higher cerebrovascular SEYLL-rates for the female population. A reversing trend was observed thereafter with male SEYLL-rates exceeding those of the female and peaking in the age-group 85+ with 2,529.9 SEYLL/100,000 compared to 1,496.8 SEYLL/100,000 for women. Lower respiratory infections (ranked 3rd) strongly affected the elderly population, reaching the maximum rates of 12,149.5 SEYLL/100,000 and 10,249.4 SEYLL/100,000 for men and women in the age-group of 85 years and older, respectively. In contrast, self-inflicted injuries strongly affected the younger population aged 15–44 years with maxima of 1,288.6 SEYLL/100,000 for men (age-group 25–29) and 493 SEYLL/100,000 for women (age-group 30–34), respectively. At later ages, these rates declined for both sexes until the age-group 65–69 but increased again with a peak at age-group 75–79 years. SEYLL-rates for colon and rectum cancers presented typical patterns for malignant neoplasms, affecting the population in the second half of the life-span with highest rates observed for the population aged 80–84, with 2,084.7 SEYLL/100,000 for males and 1,891.6 SEYLL/100,000 for females.

The majority of single leading causes (except self-inflicted injuries) showed high concentrations of SEYLL in the later stages of life.

### Time discounting and age-weighting effects

**Figure 5:**
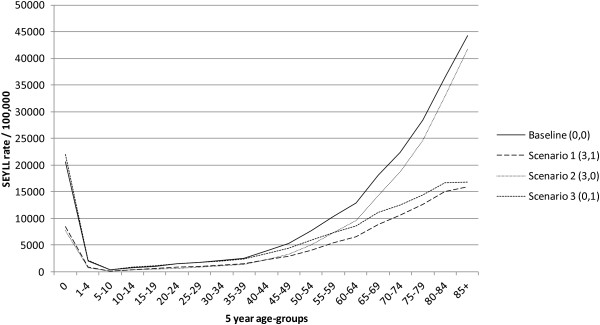
**Scenario analysis assuming different constellations of age-weighting and time-discounting.** Baseline scenario (0,0): standard SEYLL without time-discounting and uniform age-weighting. Scenario 1 (3,1): 3% time-discounting and non-uniform age-weights. Scenario 2 (3,0): 3% time-discounting and uniform age-weights. Scenario 3 (0,1): no time-discounting and non-uniform age-weights.

Three additional scenarios were used to demonstrate the effects of time-discounting and age-weighting on the disease burden due to premature death (Figure 5). Scenario one (3,1) resulted in the lowest modulated SEYLL, with a decrease of the overall disease burden by 51.6% compared to the baseline scenario (0,0). The highest decrease was observed for group I conditions, with a reduction of 55.1%. Scenario two (3,0) resulted in the highest modulated SEYLL, with an overall decrease of 25.5% as compared to the baseline scenario, with the highest reductions observed for group III conditions (−39.8%). Scenario three (0,1) showed intermediate modulated SEYLL, with a decrease of total SEYLL by 33.7% compared to the baseline estimates. The strongest reductions were found for group I conditions (−38%) (see Additional file 1 for detailed information).

Major changes in leading causes of premature death were observed for scenario three (0,1). Considering higher age-weights for the productive age groups, conditions occurring in earlier stages of life, and in particular self-inflicted injuries, received an upturn in priority ranking from 5th to 3rd position, even exceeding lower respiratory infections (data not shown).

### Local Hong Kong life-expectancy

Sensitivity analyses using local Hong Kong standard life-expectancy indicated considerable changes of SEYLL, especially for the female population (Additional file 2). Using the Hong Kong standard, female remaining life-expectancy at birth was 3.5 years longer than the WHO standard. The remaining Hong Kong life-expectancy for men was nearly identical at birth but slightly higher with increasing age. Altering the life-expectancy resulted in an increase of total SEYLL of 10.8% (15.1% for female; 7.4% for male). In particular, SEYLL due to group I conditions for the female population increased by 18.6%.

**Figure 6:**
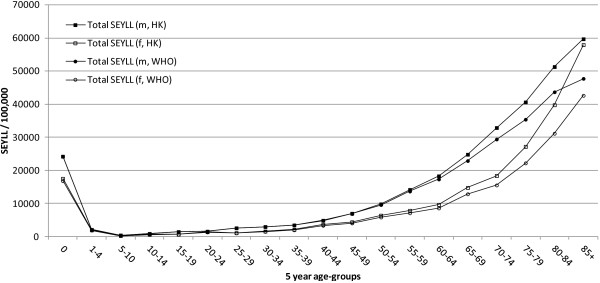
SEYLL rates for total SEYLL using Hong Kong (HK) and World Health Organisation (WHO) standard life expectancies.

The strongest impact of prolonged life-expectancy was identified for women aged 60 and older, with total SEYLL-rates per 100,000 being between 1.1 and 1.3 times higher as compared to the baseline (Figure 6). For men, the highest SEYLL-rate ratios were observed at age 70 and onwards, with SEYLL-rates being between 1.1 and 1.25 times higher than the WHO standard scenario.

### SEYLL trends over time

In total, the disease burden due to premature mortality increased remarkably between 2001 and 2010, by 6.4% (2001: 491,211 SEYLL; 2010: 524,706 SEYLL). Taking into account changes in the population (e.g. population growth), total SEYLL-rates per 100,000 population increased from 7,315.9 SEYLL/100,000 (2001) to 7,470 SEYLL/100,000 (2010). The strongest increase was observed for the male population, with a rise from 9,010.4 SEYLL/100,000 in 2001 to 9,289.1 SEYLL/100,000 in 2010. At the first level of disaggregation, an upturn of SEYLL due to group I and II conditions was identified for the male population, with SEYLL-rates for group I conditions being 1.4-fold higher in 2010 (Table 3). In contrast, a downturn of 27% of SEYLL-rates for group III conditions among males was observed. The total disease burden for women remained constant over the period. However, a considerable increase was identified for SEYLL-rates due to group I conditions, which in 2010 were 1.4-fold higher. Group II conditions remained constant and SEYLL-rates due to group III conditions decreased by 22% over the same time period.

**Table 3:** SEYLL rates per 100,000* stratified by sex and broad cause groups (2001–2010)

	**2001**	**2002**	**2003**	**2004**	**2005**	**2006**	**2007**	**2008**	**2009**	**2010**
**Total**										
**Group I**	707.3	686.9	827.8	773.6	815.9	757.9	847.4	905.0	876.1	947.7
**Group II**	5747.6	5736.6	5900.2	5851.8	5991.1	5798.4	5831.2	6017.7	5969.2	5887.3
**Group III**	861.0	900.6	924.7	835.7	740.5	711.5	688.0	712.5	682.6	635.0
**Total**	7315.9	7324.0	7652.7	7461.1	7547.5	7267.8	7366.6	7635.2	7527.9	7470.0
**Male**										
**Group I**	872.4	862.8	1014.2	920.3	1007.3	924.9	1083.3	1110.2	1088.3	1177.5
**Group II**	6951.7	6857.6	7095.0	7088.1	7231.0	7044.6	7189.3	7366.1	7304.3	7245.1
**Group III**	1186.4	1283.9	1293.8	1144.5	1007.6	957.6	959.0	928.4	923.4	866.5
**Total**	9010.4	9004.3	9403.1	9152.9	9245.9	8927.2	9231.6	9404.7	9316.0	9289.1
**Female**										
**Group I**	549.4	520.3	652.8	637.4	639.8	605.7	634.2	720.9	687.1	744.8
**Group II**	4596.2	4675.3	4778.6	4703.3	4850.9	4662.2	4603.4	4808.0	4780.1	4688.1
**Group III**	549.9	537.8	578.1	548.7	494.9	487.2	442.9	518.8	468.2	430.4
**Total**	5695.6	5733.4	6009.5	5889.5	5985.6	5755.0	5680.5	6047.8	5935.3	5863.3

Group I: communicable, maternal, perinatal and nutritional conditions.

Group II: non-communicable conditions.

Group III: injuries.

*The rates presented here are rates per 100,000 population (inhabitants of Hong Kong).

## Discussion

The results of this study represent the first comprehensive quantification of SEYLL for the population of Hong Kong. In general, Hong Kong shows a burden of disease profile that is comparable with the patterns of other regions with a high income level. Malignant neoplasms and cardiovascular diseases were identified to contribute 60.8% to the overall SEYLL of the Hong Kong population, which is consistent with studies in other high-income countries (e.g. Spain: 57.5%; Germany (North Rhine-Westphalia): 69.4%) [5,7,18,21].

Overall, SEYLL-rates for men exceeded those for women for virtually all of the ten leading causes except breast cancer, which is a sex-specific cancer with major impact on the female population. Except for injury conditions, most SEYLL were located in age-groups above 30 years, which is in line with studies from other high-income countries [7,21].

However, most of the studies identified ischaemic heart diseases as the leading single cause [5,21]. For Hong Kong, trachea, bronchus and lung cancers were the leading cause of SEYLL, which is probably associated with the still very high prevalence of smoking in Hong Kong. Although the prevalence of daily smokers had decreased from 39.7% in 1983 to 22% in 2000 for men and from 5.6% to 3.5% for women [32], the effects of past smoking habits still seem to be obvious. A recent household survey showed that the overall smoking prevalence (2009–2010) remained high, at 20.8% for men and 3.7% for women [33]. Despite the fact that smoking has been banned from many public places since 2007 [34], it is still considered a threat to the health of the population in Hong Kong. Non-smoking campaigns aiming at preventing smoking at younger ages (15–19 years), where smoking prevalence is currently low (1.8%), are important but more worrying is the much higher smoking prevalence in older age-groups, particularly in the group of 30–39 (15.6%) year-old males [33]. This observation is alarming and calls for further targeted public health campaigns to reduce the future smoking-related disease burden and health care expenditures.

At 12.7%, Hong Kong showed a considerably higher proportion of group I conditions than recent studies e.g. from Spain (6.4%) [7] or Germany (5.6%) [5]. In contrast to other high-income countries, lower respiratory infections (e.g. influenza, pneumonia) were the leading cause of SEYLL among women (e.g. ranked 9th in Spain [7]). Infectious diseases still appear to be of importance for the Hong Kong population and should be further tackled by preventive measures (e.g. promotion of influenza vaccination in the elderly population).

At 8.5% (6.9%, scenario 3,0), group III conditions showed a higher share of the total SEYLL as compared to the study from Germany (5.3%) [5] but was considerably lower than for Spain (12.9%) [7]. However, the injury patterns differed greatly as 67.3% of injury SEYLL in Hong Kong were due to intentional injury deaths. These percentages were considerably lower in the Spanish (30%) and German (35.6%) studies [5,7]. In particular, self-inflicted injuries among men aged 25–29 years alone accounted for 50.5% of the total SEYLL in this age group in Hong Kong. Studies have indicated that during this period of early adulthood both men and women are highly vulnerable to depressive symptoms due to interpersonal crisis and also have a relatively high risk of attempting or committing suicide [35].

The priorities set up by SEYLL indicated that self-inflicted injuries were ranked 6th and 7th for males and females, respectively. If the rankings were based solely on death counts, self-inflicted injuries for 2010 would not belong to the ten leading causes for women (ranked 17th) and would only be ranked 9th for men. Using SEYLL highlights the importance of self-inflicted injuries because deaths occur at an earlier stage of life and thus cause a considerable number of life years lost.

Malignant neoplasms also played an important role because this cause group accounted for about 39.1% of the total SEYLL disease burden. Trachea, bronchus and lung cancers were the leading single disease entity. However, priorities set by classical death statistics rank trachea, bronchus and lung cancers only in 3rd place. When not considering age at death, priorities are shifted towards lower respiratory infections (ranked 1st) and ischaemic heart diseases (ranked 2nd), conditions with major impact on the elderly population. The use of SEYLL-rates compared to standard death counts drew greater attention not only to breast cancer for women (whose rank changed from 8th to 5th) but also to liver cancer for men (whose rank changed from 7th to 4th).

As shown in the scenario analyses, the choice of social values (time-discounting and age-weighting) can have a large impact on the resulting SEYLL, with reductions of up to half of the total SEYLL. Previous studies presented SEYLL with a 3% time-discounting and uniform age-weighting and thus especially depleted the health losses of conditions occurring in earlier stages of life [5,7]. In particular, SEYLL due to group III conditions, including self-inflicted injuries, were reduced by 39.8% when a 3% time-discounting was applied.

The effects of replacing the WHO’s standards with the local Hong Kong life-expectancy values mainly impacted on the SEYLL-rates of women and the biggest overall changes were observed for older age-groups because most of the deaths are condensed in this life-span. For the sex-specific leading causes of SEYLL, however, the rankings were almost identical (Additional file 3).

The time-series analyses highlighted the trend of increasing disease burden, especially for group I conditions. Lower respiratory infections were one of the major drivers of SEYLL in Hong Kong and intervention measures should therefore focus on target groups of the population aged 55 and older.

The present study is founded solely on mortality data, which may be deficient in capturing the full impact of diseases and injuries. Including the morbidity component in further assessments is important because, particularly in high-income countries, the share of chronic diseases is increasing dramatically, as indicated by the GBD study results for the years 2004 and 2010 [12,36]. Further, possible adjustment of the GBD classification system towards the needs of the disease patterns observed in Hong Kong may lead to more detailed results as e.g. the category of “other malignant neoplasms”, which could further be disaggregated, has high numbers of SEYLL and may mask single important entities.

This study has demonstrated that SEYLL is a valuable measure to quantify the impact of premature deaths on population health because it takes into account the potentially remaining life-expectancy at age of death [18,21]. Another frequently applied measure of premature mortality, the potential years of life lost (PYLL) (used e.g. by the Organisation for Economic Co-operation and Development), use potential limits to life (e.g. 75 years). This method does not take into account lost years due to deaths occurring at ages above this limit [15,37-39]. Thus, interventions aiming at avoiding deaths above 75 years would result in no benefit. The SEYLL measure uses remaining life-expectancy values and takes into account deaths in the elderly population and thus is more sensitive to deaths occurring in this life-span [39]. Taking into account the age of death and the remaining life-expectancy, the SEYLL offers a more appropriate measure of premature death.

## Conclusions

Using the SEYLL as a measure of premature mortality allows considering the age at death and thus represents a highly qualified measure of mortality as compared to traditionally used mortality indicators such as simple death counts. Especially conditions that occur in earlier stages of life are captured more adequately which results in significant changes in priorities. In our study we identified “trachea, bronchus and lung cancers” as the major cause of SEYLL in Hong Kong in the year 2010 probably associated with still high smoking rates in adults. As SEYLL only represents one component of the overall disease burden as measured by the DALY further analyses should aim at estimating the morbidity component (YLD) to allow a more comprehensive estimation of the disease burden for Hong Kong. Including the morbidity perspective would have a high impact on disease patterns and may lead to changes in priority setting.

## Competing interests

The authors declared that they have no competing interests.

## Authors’ contributions

DP conceptualized the study, performed the analyses, interpreted the results and drafted the manuscript. PYKC gathered and harmonized the data, interpreted the results and critically revised the manuscript for important intellectual content. TQT, HJJ, PCL, and CMW interpreted the results and critically revised the manuscript for important intellectual content. AK conceptualized the study, advised on the analyses, interpreted the results and critically revised the manuscript for important intellectual content. All authors read and approved the final version of the manuscript.

## Pre-publication history

The pre-publication history for this paper can be accessed here:

http://www.biomedcentral.com/1471-2458/13/863/prepub

## Supplementary Material

Additional file 1 Scenario analysis assuming different time-discounting and age-weighting assumptions. Click here for file

Additional file 2 Comparison between Hong Kong and WHO standard life expectancy. Click here for file

Additional file 3 Alteration of disease priorities due to changes of life expectancy (LE). Click here for file

## References

[B1] Horton R The global financial crisis: an acute threat to health Lancet 2009 373 355 356 10.1016/S0140-6736(09)60116-1 19186260

[B2] Marmot MG Bell R How will the financial crisis affect health? BMJ 2009 338 858 860 10.1136/bmj.b858 19359306

[B3] Chuang Y-C Chuang K-Y Chen Y-R Shi B-W Yang T-H Welfare state regimes, infant mortality and life expectancy: integrating evidence from east Asia JECH 2012 66 e23 10.1136/jech.2010.126961 21880645

[B4] Cheung KS Yip PS Trends in healthy life expectancy in Hong Kong SAR 1996–2008 Eur J Ageing 2010 7 257 269 10.1007/s10433-010-0171-3 21212818 PMC2995861

[B5] Penner D Pinheiro P Krämer A Measuring the burden of disease due to premature mortality using standard expected years of life lost (SEYLL) in North Rhine-Westphalia, a federal state of Germany, in 2005 JPH 2010 18 319 325

[B6] Marshall RJ Standard expected years of life lost as a measure of mortality: norms and reference to New Zealand data Aust N Z J Public Health 2004 28 452 457 15707187 10.1111/j.1467-842x.2004.tb00027.x

[B7] Genova-Maleras R Catala-Lopez F de Larrea-Baz N Alvarez-Martin E Morant-Ginestar C The burden of premature mortality in Spain using standard expected years of life lost: a population-based study BMC Public Health 2011 11 787 10.1186/1471-2458-11-787 21989453 PMC3208248

[B8] Michaud C McKenna M Begg S Tomijima N Majmudar M Bulzacchelli M Ebrahim S Ezzati M Salomon J Gaber Kreiser J The burden of disease and injury in the United States 1996 Popul Health Metr 2006 4 11 10.1186/1478-7954-4-11 17049081 PMC1635736

[B9] Lopez AD Mathers CD Ezzati M Jamison DT Murray CJL Global and regional burden of disease and risk factors, 2001: systematic analysis of population health data Lancet 2006 367 1747 1757 10.1016/S0140-6736(06)68770-9 16731270

[B10] Dodhia H Phillips K Measuring burden of disease in two inner London boroughs using disability adjusted life years J Public Health 2008 30 3 313 321 fdn015 10.1093/pubmed/fdn015 18400697

[B11] Zhou SC Cai L Wang J Cui SG Chai Y Liu B Wan CH Measuring the burden of disease using disability-adjusted life years in Shilin county of Yunnan province, China Environ Health Prev Med 2011 16 148 154 10.1007/s12199-010-0176-8 21431803 PMC3078293

[B12] WHO The global burden of disease: 2004 update 2008 Geneva: World Health Organization

[B13] Field MJ Gold MR Summarizing population health directions for the development and application of population metrics Summarizing population health directions for the development and application of population metrics 1998 Washington: National Academy Press 85 25101456

[B14] Murray CJL Lopez AD World Health Organization, World Bank, Harvard School of Public Health The global burden of disease: a comprehensive assessment of mortality and disability from diseases, injuries, and risk factors in 1990 and projected to 2020: summary 1996 Geneva: World Health Organization

[B15] Murray CJL Quantifying the burden of disease: the technical basis for disability-adjusted life years Bull World Health Organ 1994 72 429 445 8062401 PMC2486718

[B16] Boerma JT Stansfield SK Health statistics now: are we making the right investments? Lancet 2007 369 779 786 10.1016/S0140-6736(07)60364-X 17336655

[B17] Fontaine KR Redden DT Wang C Westfall AO Allison DB Years of life lost due to obesity JAMA 2003 289 187 193 10.1001/jama.289.2.187 12517229

[B18] Mariotti S D’Errigo P Mastroeni S Freeman K Years of life lost due to premature mortality in Italy Eur J Epidemiol 2003 18 513 521 12908716 10.1023/a:1024635401206

[B19] Allard YE Wilkins R Berthelot JM Premature mortality in health regions with high aboriginal populations Health Rep 2004 15 51 60 14999891

[B20] Burnet NG Jefferies SJ Benson RJ Hunt DP Treasure FP Years of life lost (YLL) from cancer is an important measure of population burden and should be considered when allocating research funds Br J Cancer 2005 92 241 245 15655548 10.1038/sj.bjc.6602321 PMC2361853

[B21] Vlajinac H Marinkovic J Kocev N Sipetic S Bjegovic V Jankovic S Stanisavljevic D Markovic-Denic L Maksimovic J Years of life lost due to premature death in Serbia (excluding Kosovo and Metohia) Public Health 2008 122 277 284 10.1016/j.puhe.2007.06.010 17825856

[B22] Lopez AD Global and regional burden of disease and risk factors, 2001: systematic analysis of population health data Lancet 2006 367 1747 10.1016/S0140-6736(06)68770-9 16731270

[B23] Mathers CD Vos T Salomon J Ezzati M National burden of disease studies: a practical guide 2001 Geneva: World Health Organization

[B24] Hong Kong Census and Statistics Department Hong Kong annual digest of statistics Hong Kong annual digest of statistics 2011 2011 Hong Kong:

[B25] Anand S Hanson K DALYs: efficiency versus equity World Dev 1998 26 307 310 10.1016/S0305-750X(97)10019-5

[B26] Anand S Hanson K Disability-adjusted life years: a critical review J Health Econ 1997 16 685 702 10.1016/S0167-6296(97)00005-2 10176779

[B27] Arnesen T Kapiriri L Can the value choices in DALYs influence global priority-setting? Health Policy 2004 70 137 149 10.1016/j.healthpol.2003.08.004 15364144

[B28] HK SAR Government Department Hong Kong yearbook 2010 2010 Hong Kong: the Goverment Logistics Department

[B29] Hong Kong Census and Statistics Department Hong Kong SAR: 2001–2010 known death microdata sets Hong Kong SAR: 2001–2010 known death microdata sets, 21 december 2011 edition 2011 Hong Kong

[B30] Lopez AD Disease Control Priorities Project Global burden of disease and risk factors 2006 New York, NY, Washington, DC: Oxford University Press, World Bank

[B31] Lozano R Murray CJL Lopez AD Satoh T World Health Organization Miscoding and misclassification of ischaemic heart disease mortality 2001 Geneva: World Health Organization

[B32] Au JSK Mang OWK Foo W Law SCK Time trends of lung cancer incidence by histologic types and smoking prevalence in Hong Kong 1983–2000 Lung Cancer 2004 45 143 152 10.1016/j.lungcan.2004.01.012 15246184

[B33] HK SAR Census and Statistics Department Thematic houshold survey report No. 48 Thematic houshold survey report No. 48 2011 Hong Kong: Social Surveys Section Census and Statistics Department

[B34] Smoking (public health) ordinance http://www.legislation.gov.hk/blis_pdf.nsf/6799165D2FEE3FA94825755E0033E532/FC20BFF93D75ADCC482575EE00764F46/$FILE/CAP_371_e_b5.pdf

[B35] Shah A The relationship between suicide rates and age: an analysis of multinational data from the world health organization Int Psychogeriatr 2007 19 1141 1152 17433118 10.1017/S1041610207005285

[B36] Murray CJL Vos T Lozano R Naghavi M Flaxman AD Michaud C Ezzati M Shibuya K Salomon JA Abdalla S Disability-adjusted life years (DALYs) for 291 diseases and injuries in 21 regions, 1990–2010: a systematic analysis for the global burden of disease study 2010 Lancet 2012 380 2197 2223 10.1016/S0140-6736(12)61689-4 23245608

[B37] OECD Health at a glance 2011: OECD indicators 2011 OECD Publishing http://dx.doi.org/10.1787/health_glance-2011-en

[B38] Werber D Hille K Frank C Dehnert M Altmann D Muller-Nordhorn J Koch J Stark K Years of potential life lost for six major enteric pathogens, Germany, 2004–2008 Epidemiol Infect 2012 141 1 8 22813426 10.1017/S0950268812001550 PMC9151843

[B39] Murray CJL Mathers CD Salomon JA Lopez AD Murray CJL, Salomon JA, Mathers CD, Lopez AD Health gaps: an overview and critical appraisal Summary measures of population health - concepts, ethics, measurement and applications 2002 Geneva: World Health Orgnaization 233 244

