# Supplementary material for: Quantifying the burden of disease due to premature mortality in Hong Kong using standard expected years of life lost
Source: BMC Public Health. 2013 Sep 18;13:863. doi: 10.1186/1471-2458-13-863 (PMC3848717; doi:10.1186/1471-2458-13-863)
Supplement: Additional file 1 — Scenario analysis assuming different time-discounting and age-weighting assumptions. [file 1471-2458-13-863-S1.docx]

| *Additional file 1: Scenario analysis assuming different time-discounting and age-weighting assumptions* | | | | | | | |
| --- | --- | --- | --- | --- | --- | --- | --- |
|  |  |  |  |  |  |  |  |
| **Scenario** | **0,0** | **3,1** | **SEYLL change** | **3,0** | **SEYLL change** | **0,1** | **SEYLL change** |
| **Group I** | 66568.5 | 29914.3 | -55.1 | 51420.9 | -22.8 | 41301.4 | -38.0 |
| **Group II** | 413537.0 | 199749.3 | -51.7 | 312575.6 | -24.4 | 267799.0 | -35.2 |
| **Group III** | 44600.9 | 24132.8 | -45.9 | 26847.1 | -39.8 | 38528.0 | -13.6 |
| **Total** | 524706.5 | 253796.4 | -51.6 | 390843.6 | -25.5 | 347628.4 | -33.7 |

Group I: communicable, maternal, perinatal and nutritional conditions

Group II: non-communicable conditions

Group III: injuries
