# Supplementary material for: Quantifying the burden of disease due to premature mortality in Hong Kong using standard expected years of life lost
Source: BMC Public Health. 2013 Sep 18;13:863. doi: 10.1186/1471-2458-13-863 (PMC3848717; doi:10.1186/1471-2458-13-863)
Supplement: Additional file 2 — Comparison between Hong Kong and WHO standard life expectancy. [file 1471-2458-13-863-S2.docx]

| Additional file 2: Comparison between Hong Kong and WHO standard life expectancy | | | | | | | | | | | | | | |
| --- | --- | --- | --- | --- | --- | --- | --- | --- | --- | --- | --- | --- | --- | --- |
|  | Hong Kong | | |  | WHO | |  | | Difference in years | | |  |  |  |
| age-group | | male | female | | | male | | female | | male | female | | |  |
| 0 | | 80.0 | 85.9 | | | 79.9 | | 82.4 | | 0.0 | 3.5 | | |  |
| 1-4 | | 77.7 | 83.6 | | | 77.8 | | 80.3 | | -0.1 | 3.3 | | |  |
| 5-9 | | 72.8 | 78.7 | | | 72.9 | | 75.5 | | -0.1 | 3.2 | | |  |
| 10-14 | | 67.8 | 73.7 | | | 67.9 | | 70.5 | | -0.1 | 3.2 | | |  |
| 15-19 | | 62.9 | 68.8 | | | 62.9 | | 65.6 | | 0.0 | 3.2 | | |  |
| 20-24 | | 58.0 | 63.8 | | | 58.0 | | 60.6 | | 0.0 | 3.2 | | |  |
| 25-29 | | 53.1 | 58.9 | | | 53.0 | | 55.7 | | 0.1 | 3.2 | | |  |
| 30-34 | | 48.2 | 53.9 | | | 48.0 | | 50.8 | | 0.2 | 3.1 | | |  |
| 35-39 | | 43.4 | 49.0 | | | 43.1 | | 46.0 | | 0.3 | 3.1 | | |  |
| 40-44 | | 38.6 | 44.2 | | | 38.2 | | 41.1 | | 0.4 | 3.1 | | |  |
| 45-49 | | 33.9 | 39.4 | | | 33.4 | | 36.4 | | 0.5 | 3.0 | | |  |
| 50-54 | | 29.3 | 34.7 | | | 28.7 | | 31.7 | | 0.7 | 3.0 | | |  |
| 55-59 | | 24.9 | 30.0 | | | 24.1 | | 27.1 | | 0.9 | 2.9 | | |  |
| 60-64 | | 20.8 | 25.5 | | | 19.7 | | 22.6 | | 1.1 | 2.8 | | |  |
| 65-69 | | 16.8 | 21.1 | | | 15.5 | | 18.3 | | 1.3 | 2.7 | | |  |
| 70-74 | | 13.3 | 16.9 | | | 11.9 | | 14.2 | | 1.4 | 2.6 | | |  |
| 75-79 | | 10.1 | 13.0 | | | 8.8 | | 10.6 | | 1.3 | 2.4 | | |  |
| 80-84 | | 7.5 | 9.6 | | | 6.3 | | 7.6 | | 1.1 | 2.1 | | |  |
| 85+ | | 4.4 | 5.8 | | | 3.5 | | 4.3 | | 0.9 | 1.5 | | |  |
