# Supplementary material for: Quantifying the burden of disease due to premature mortality in Hong Kong using standard expected years of life lost
Source: BMC Public Health. 2013 Sep 18;13:863. doi: 10.1186/1471-2458-13-863 (PMC3848717; doi:10.1186/1471-2458-13-863)
Supplement: Additional file 3 — Alteration of disease priorities due to changes of life expectancy (LE). [file 1471-2458-13-863-S3.docx]

| Additional file 3: Alteration of disease priorities due to changes of life expectancy (LE) | | | | | | | | | | | | | | | | | | | |
| --- | --- | --- | --- | --- | --- | --- | --- | --- | --- | --- | --- | --- | --- | --- | --- | --- | --- | --- | --- |
| **WHO (LE)** | | |  |  | | **Hong Kong (LE)** | | | | | | | | | | | | |  |
| *Total* | | | |  | |  | | |  | | | | | |  | | | | |
| Condition | Rank | SEYLL | |  | | | Rank | | | | | SEYLL | | | | |  |  |  |
| Trachea, bronchus, lung cancers | 1 | 52242.1 | |  | | | 2 | | | | | 57929.7 | | | | |  |  |  |
| Ischaemic heart disease | 2 | 51542.2 | |  | | | 1 | | | | | 58742.3 | | | | |  |  |  |
| Lower respiratory infections | 3 | 46503.2 | |  | | | 3 | | | | | 54950.3 | | | | |  |  |  |
| Cerebrovascular disease | 4 | 35970.7 | |  | | | 4 | | | | | 41360.0 | | | | |  |  |  |
| Self-inflicted injuries | 5 | 29524.9 | |  | | | 6 | | | | | 30920.3 | | | | |  |  |  |
| Colon and rectum cancers | 6 | 27833.3 | |  | | | 5 | | | | | 31269.8 | | | | |  |  |  |
| Liver cancer | 7 | 25796.8 | |  | | | 7 | | | | | 27973.9 | | | | |  |  |  |
| Other malignant neoplasms | 8 | 16392.2 | |  | | | 8 | | | | | 17965.6 | | | | |  |  |  |
| Nephritis and nephrosis | 9 | 15172.3 | |  | | | 9 | | | | | 17593.7 | | | | |  |  |  |
| Breast cancer | 10 | 14544.2 | |  | | | 11 | | | | | 16209.9 | | | | |  |  |  |
|  |  | | |  | |  | | |  | | | | | |  | | | | |
| *Female* | | | |  | |  | | |  | | | | | |  | | | | |
| Condition | Rank | SEYLL | | |  | | | | | Rank | | | SEYLL | | |  |  |  |  |
| Lower respiratory infections | 1 | 19733.81 | | |  | | | | | 1 | | | 24847.9 | | |  |  |  |  |
| Ischaemic heart disease | 2 | 18073.74 | | |  | | | | | 2 | | | 22277.8 | | |  |  |  |  |
| Trachea, bronchus, lung cancers | 3 | 17907.28 | | |  | | | | | 3 | | | 20781.8 | | |  |  |  |  |
| Cerebrovascular disease | 4 | 16790.58 | | |  | | | | | 4 | | | 20331.3 | | |  |  |  |  |
| Breast cancer | 5 | 14464.52 | | |  | | | | | 5 | | | 16125.4 | | |  |  |  |  |
| Colon and rectum cancers | 6 | 12279.83 | | |  | | | | | 6 | | | 14409.6 | | |  |  |  |  |
| Self-inflicted injuries | 7 | 11647.5 | | |  | | | | | 7 | | | 12649.2 | | |  |  |  |  |
| Other malignant neoplasms | 8 | 7342.277 | | |  | | | | | 10 | | | 8379.8 | | |  |  |  |  |
| Nephritis and nephrosis | 9 | 7273.759 | | |  | | | | | 8 | | | 8841.9 | | |  |  |  |  |
| Other cardiovascular diseases | 10 | 7118.351 | | |  | | | | | 9 | | | 8685.7 | | |  |  |  |  |
|  |  | | |  | |  | | |  | | | | | |  | | | | |
| *Male* |  | | |  | |  | | |  | | | | | |  | | | | |
| Condition | Rank | SEYLL | | | | | |  | | | Rank | | | SEYLL | | | |  |  |
| Trachea, bronchus, lung cancers | 1 | 34334.82 | | | |  | | | | 1 | | | 37148 | | |  |  |  |  |
| Ischaemic heart disease | 2 | 33468.49 | | | |  | | | | 2 | | | 36464 | | |  |  |  |  |
| Lower respiratory infections | 3 | 26769.38 | | | |  | | | | 3 | | | 30102 | | |  |  |  |  |
| Liver cancer | 4 | 19470.44 | | | |  | | | | 5 | | | 20649 | | |  |  |  |  |
| Cerebrovascular disease | 5 | 19180.07 | | | |  | | | | 4 | | | 21029 | | |  |  |  |  |
| Self-inflicted injuries | 6 | 17877.41 | | | |  | | | | 6 | | | 18271 | | |  |  |  |  |
| Colon and rectum cancers | 7 | 15553.44 | | | |  | | | | 7 | | | 16860 | | |  |  |  |  |
| Chronic obstructive pulmonary disease | 8 | 11794.83 | | | |  | | | | 8 | | | 13403 | | |  |  |  |  |
| Mouth and oropharynx cancers | 9 | 9246.028 | | | |  | | | | 9 | | | 9663.6 | | |  |  |  |  |
| Other malignant neoplasms | 10 | 9049.938 | | | |  | | | | 10 | | | 9585.8 | | |  |  |  |  |
